# American Medical Association

**Published:** 1876-07

**Authors:** 


*American Medical Association.*—We did not go to Philadelphia to attend the Association, having business that prevented a regular attendance upon its meetings; yet we visited that body as frequently as possible and noted perhaps everything of special interest that was done. Touching the question of our friend in regard to the failure of the Association to accomplish any good in the past, we have to remark that, while it has not met the expectations of its founders fully, it has, nevertheless, published many able and important essays, and contributed largely to American medical literature. That it has accomplished little or nothing towards the elevation of the profession in important matters of medical education, we freely concede. Nor has it regulated and dignified the conduct of the schools in the matter of conferring degrees, or exerted, so far as can be seen, that controlling influence upon the moral and ethical deportment of the members of the profession in this country that was anticipated.

The late meeting, like those that preceded it, took no position or binding action in regard to the colleges; yet, I was glad to note a decided and strong undercurrent in favor of a radical and important advancement in this direction, and we could not but feel gratified that the views of our journal, so often and so strongly urged in favor of elevating the standard of medical education, were entertained by large numbers, and there were indications that at no distant day great and important changes in the matter of medical education will be demanded of the colleges in this country. In this connection, we will allude to the Convention of Medical Colleges which took place on the 2d of June, four days in advance of the American Medical Association. Here, while the same undercurrent of a desire to take an upward step in the cause of medical education prevailed, yet nothing definite or binding was done. Important questions were presented and discussed. Resolutions were passed condemning the beneficiary and short term systems, and striking at those schools which have adopted them. The double school at Louisville and the Long Island Medical College were doubtless aimed at. The Michigan University received, also, a blow in the following action:

*Resolved*, That in the opinion of this Association, Medical Colleges ought not to recognize or hold fellowship with any school or alumni in which irregular medicine is taught as part of the curriculum.

We presume that our readers are aware that the trustees of that institution recognized Homœpathy; and it will be remembered that we denounced strongly this action through our journal at the time it was done. We did not then know that it was done without the approval or consent of the faculty—a fact which we learned in Philadelphia. It seems, therefore, unjust that the faculty should be denounced, and so long as they oppose and resist such action on the part of the trustees, they are entitled to the sympathy rather than the condemnation of the profession. While the University as a whole cannot be recognized, the original faculty, it would appear, ought not to be held responsible, but should, we think, be upheld and sustained by the profession, in the hope that the stand taken by this Convention and the moral influence thereby exerted, may open the eyes of the trustees to the impropriety of their action in the matter, and lead to its early abolishment.

While these important resolutions were adopted by the Convention, indicating the existence of the proper spirit in that body, yet the following resolution destroys in a large degree the satisfaction which we would otherwise have felt:

*Resolved*, That the action of the Convention shall not be considered binding upon the college represented, unless endorsed by their respective faculties.

Thus our friend will perceive that nothing binding or definite was done by the Convention. For this reason it has been regarded by many as a farce, and in one sense it was. But it should be remembered that it was a provisional association, and hence its action was mere advisory. If, however, it shall hereafter become a permanent organization, we may reasonably hope that a great work of reform will be brought about, and that the sentiment of those in the profession who advocate a higher grade of education and a *more honorable* management of these institutions will not only be respected, but enforced. The alumni, who annually go forth from the schools, are expected to observe the ethics, if they would obtain recognition from the high toned and honorable among the profession. So the time will, or ought to come, when the schools themselves, who are in any sense irregular and who disregard the ethics, either in in spirit or letter, will be ostracized by their fellows, and condemned by the profession at large.

Where is the propriety or right on the part of the colleges to resort to undercutting in rates, or other irregularity to obtain patronage, any more than the practitioner can resort to like empirical procedure, to obtain a dishonorable success over his fellows? The opinion is rapidly gaining strength among the members of the profession, that such right does not pertain to the colleges, and that the conduct of several of our schools in this matter is unfair, and utterly violative of both the letter and spirit of the ethics. Whilst we thus freely and candidly express our views upon this subject, yet we can well understand and appreciate the obstacles which have heretofore prevented the colleges from adopting the needed reforms, and having confered personally with many of them at the convention, we feel greatly encouraged at the prospect held up for the future, in regard to this important subject.

